# Family History, Diabetes, and Other Demographic and Risk Factors Among Participants of the National Health and Nutrition Examination Survey 1999–2002

**Published:** 2005-03-15

**Authors:** Ann M Annis, Mark S Caulder, Michelle L Cook, Debra Duquette

**Affiliations:** Genomics, Michigan Department of Community Health; Michigan Department of Community Health, Lansing, Mich; Michigan Department of Community Health, Lansing, Mich; Michigan Department of Community Health, Lansing, Mich

## Abstract

**Introduction:**

Family history of diabetes has been recognized as an important risk factor of the disease. Family medical history represents valuable genomic information because it characterizes the combined interactions between environmental, behavioral, and genetic factors. This study examined the strength and effect of having a family history of diabetes on the prevalence of self-reported, previously diagnosed diabetes among adult participants of the National Health and Nutrition Examination Survey 1999–2002.

**Methods:**

The study population included data from 10,283 participants aged 20 years and older. Gender, age, race/ethnicity, poverty income ratio, education level, body mass index, and family history of diabetes were examined in relation to diabetes status. Diabetes prevalence estimates and odds ratios of diabetes were calculated based on family history and other factors.

**Results:**

The prevalence of diabetes among individuals who have a first-degree relative with diabetes (14.3%) was significantly higher than that of individuals without a family history (3.2%), corresponding to a crude odds ratio of five. Both prevalence and odds ratio estimates significantly increased with the number of relatives affected with diabetes. Family history was also associated with several demographic and risk factors.

**Conclusion:**

Family history of diabetes was shown to be a significant predictor of diabetes prevalence in the adult U.S. population. We advocate the inclusion of family history assessment in public health prevention and screening programs as an inexpensive and valuable source of genomic information and measure of diabetes risk.

## Introduction

Diabetes mellitus presents multiple challenges to public health. An estimated 18.2 million individuals in the United States have diabetes (1). This disease contributes to significant morbidity, including cardiovascular, cerebrovascular, and renal disease, and premature mortality (1-3). In 2002, diabetes was ranked as the sixth leading cause of death (1,4). Another major public health challenge is the increasing prevalence of type 2 diabetes in adults, children, and adolescents during the past two decades (5-7). Additionally, type 2 diabetes may account for 90% to 95% of all diagnosed cases of diabetes (1,6,8), may progress undetected for years, and is often not diagnosed until onset of clinical symptoms or complications (3,6,8).

Undiagnosed diabetes constitutes approximately 29.3% of total diabetes prevalence (5). It is clear that developing strategies to screen and identify high-risk individuals should be an important public health goal. Screening for type 2 diabetes is recommended for individuals aged 45 years and older and/or younger individuals who have one or more risk factors, such as race/ethnicity (i.e., African American, Native American, and Hispanic), overweight or obesity, physical inactivity, previous history of gestational diabetes, and family history of diabetes (9). A primary goal of tailored screening is to recognize high-risk individuals in the presymptomatic stage of diabetes. Research has indicated that diabetes and many of its health complications can be delayed or prevented through medical and lifestyle interventions, such as pharmaceuticals, diet, and exercise (6,10-17).

For prevention efforts to be most effective, public health programs must recognize the factors involved in diabetes susceptibility. Evidence for a strong genetic element of type 2 diabetes susceptibility is suggested by the high incidence in certain racial/ethnic populations (1,3,6,18,19), high concordance in monozygotic twins compared with dizygotic twins (6,20,21), and high incidence among first-degree relatives of persons with type 2 diabetes (3,6,19,22-25). The complex pathophysiologic nature of diabetes supports the idea that multiple biologic and/or chemical pathways are implicated in the development and progression of the disease (26), and numerous genetic loci have been investigated in the search for genetic determinants of the disease (26-30). Identifying susceptibility loci for diabetes, however, has been difficult because of the multiple genes involved and strong environmental contributing factors (26).

Family history of type 2 diabetes is recognized as an important risk factor of the disease (3,6,9,19,22-25). Individuals who have a family history of diabetes can have two to six times the risk of type 2 diabetes compared with individuals with no family history of the disease (6,19). The etiologies of type 2 diabetes are complex: family medical history provides valuable genomic information because it represents the combination of inherited genetic susceptibilities and shared environmental and behavioral factors (31). The use of family history as part of a comprehensive risk assessment for an individual can be crucial in the prevention, early detection, and treatment of type 2 diabetes. On a population level, family history may help tailor health promotion messages for specific population groups (31).

A goal of this study was to assess the feasibility of obtaining and using genomic information from an existing, national population-based data source to provide chronic disease program recommendations. Specifically, our objective was to examine the strength and effect of having a family history of diabetes in first-degree relatives on the prevalence of self-reported, physician-diagnosed diabetes among adult participants in the National Health and Nutrition Examination Survey (NHANES) during 1999 to 2002. We evaluated several risk factors influencing diabetes prevalence in the United States and how these factors relate to family history.

## Methods

### Population

The National Center for Health Statistics (NCHS), within the Centers for Disease Control and Prevention (CDC), annually conducts NHANES, a continuous, population-based survey of the civilian, noninstitutionalized U.S. population (32). Data for NHANES is collected from U.S. households using two methods: an in-home interview and a physical health examination. Written informed consent is obtained from each participant for both parts of the survey. Information gathered by NHANES is intended for health research purposes, and NHANES documentation and codebooks are provided elsewhere (32).

For the study, data sets from both NHANES 1999–2000 and NHANES 2001–2002 were merged to create a NHANES 1999–2002 data set (n = 21,004) (32). Information on family history of diabetes was not available for participants aged 19 years and younger. Because family history was considered an important predictor of diabetes status, and the main focus was type 2 diabetes, subjects under the age of 20 years (n = 10,713) were excluded from the data set.

### Diabetes status

Diabetes status was self-reported by asking whether an individual had ever been told by a doctor or health professional that he/she had diabetes or "sugar diabetes" other than during pregnancy (for female respondents). Because this survey question precluded gestational diabetes, pregnant women (n = 603) were not excluded from the study. The interview process did not discriminate between type 1 and type 2 diabetes. Survey participants from whom diabetes status was not ascertained during the NHANES interview were excluded from this study (n = 8). Among the remaining 10,283 adult respondents, 991 were categorized as having diabetes (including eight pregnant females), and 9292 were categorized as not having diabetes.

Individuals who reported a previous diagnosis of diabetes were asked at which age their diagnosis occurred. Age of diagnosis information was missing for 10 subjects in the sample population. There were 83 subjects who reported an age of diagnosis younger than 20 years. Although type 1 diabetes typically occurs during these younger ages, there was no definitive way to differentiate between type 1 and type 2 diabetes, and therefore we did not exclude any subject based on age of diabetes diagnosis.

### Demographics and risk factors

Sex, age, and race were self-reported during the survey interview. Age was recorded as the subject's age in years at the time of interview. The age categories were 20–39 years, 40–59 years, and 60 years and older (33). Race and ethnicity were categorized in the following groups: non-Hispanic white, non-Hispanic black, Mexican American, and "other," which consisted of all other individual and multiracial groups. Statistical results for the "other" category are not described because the wide variability within the group prevents meaningful interpretation of estimates.

Socioeconomic status was assessed by poverty income ratio (PIR) and education level of the participants. The PIR, based on family size, is the ratio of family income to the family's poverty threshold level, determined by the Bureau of the Census (34). NHANES calculated participants' PIR values using self-reported family income data. We used the following categories: PIR <1.00, PIR 1.00–1.85, and PIR ⩾1.86. PIR values less than 1.00 are deemed to be below the poverty threshold. Some federally funded food assistance programs have an eligibility cut point of 1.85 (33,34). Education level was self-reported as the highest level achieved and was categorized as less than high school, high school or general equivalency diploma (GED), and more than high school.

During the NHANES physical examination, survey participants had both standing height (m) and weight (kg) measured, which were used to calculate body mass index (BMI [kg/m^2^]). Healthy weight was defined as BMI <25, overweight as BMI 25–29, and obesity as BMI ⩾30. Individuals who did not undergo a physical exam or who had missing BMI information and all women who were reported as being pregnant at the time of interview were excluded from analyses that contained BMI.

### Family history

Participants were asked whether any biological member of their family, living or deceased, had ever been told he/she had diabetes. Family history information was not available from 216 individuals because of participant refusal (n = 2) and lack of knowledge of family medical history (n = 214). Subjects specified the relationship of any family member with diabetes; however, diabetes in children of the participants was not ascertained. We defined family history as having a first-degree relative (parent and/or sibling) with diabetes and categorized subjects according to parental and sibling diabetes status and number of first-degree relatives with diabetes.

### Statistics

Statistical analyses were conducted using SAS version 9.1.3 (SAS Institute Inc, Cary, NC). This newest version permits analyses of complex survey designs. To achieve sufficient sample sizes, NHANES oversamples certain populations (33,34); thus, appropriate NHANES sample weights, stratums, and primary sampling units (PSUs) were used to account for complex sampling design, differential probabilities of selection, and nonresponse. Poststratification adjustments were applied by NHANES to the sample weights based on census population controls (33-35).

Prevalence estimates for diabetes, stratified by demographics and risk factors, were calculated using NHANES sampling weights and are extrapolated to the adult, noninstitutionalized, civilian U.S. population. Comparisons of diabetes prevalence between different groups were performed using F tests based on design-adjusted Rao–Scott chi squares (χ^2^). Age-adjusted prevalence (not shown) for the gender–race groups, based on the standard U.S. Census 2000 population (36), were deemed unreliable because of large associated standard errors and small sample sizes, especially in the group aged 20–39 years. For subjects with diabetes, the average age at diagnosis was examined by demographic and risk factors.

Crude and adjusted odds ratios (OR) and 95% confidence intervals (CI) for diabetes associated with family history were calculated through logistic regression analyses, which modeled the binary outcome of diabetes status (yes/no). Individual Wald χ^2^ tests and *P* values for all β estimates were computed. Four regression models were developed to first analyze family history independently, then in combination with demographic and risk factors demonstrating significant association with diabetes status. Variance estimates and standard errors were calculated using the Taylor expansion method. Any estimate with a relative standard error greater than 30% was considered to be statistically unreliable. Significance testing of interaction terms was performed to assess potential interaction between the factors included in the models. Likelihood ratio tests, multivariate Wald χ^2^ tests, and F tests were calculated to test for overall model significance. All *P* values less than .05 were considered statistically significant.

## Results

### Demographics and risk factors

The frequencies and weighted percentages of adults with diabetes are stratified by demographic and risk factors (Table 1). The overall estimated prevalence of diabetes among adults, representative of the civilian U.S. population, was 6.5%. Among men, the diabetes prevalence of non-Hispanic black men was significantly higher than that of Mexican American men (*P* = .01). Non-Hispanic black women had the highest prevalence of diabetes (11.4%) among all gender–race groups. The diabetes prevalence of non-Hispanic black women was significantly higher when compared to the prevalence of non-Hispanic white women (*P* < .001) and Mexican American women (*P* = .007). Mexican American women had significantly higher diabetes prevalence than non-Hispanic white women (*P* = .004).

The prevalence of diabetes significantly increased with age at interview (*P* = .001), and individuals 60 years and older experienced the highest prevalence (15.1%). Among the three PIR categories, adults in the group with the highest PIR level had significantly lower diabetes prevalence than adults at poverty level (*P* = .008) and in the middle PIR category (*P* < .001). Additionally, adults with less than a high school education experienced significantly higher diabetes prevalence than both those with a high school education (*P <* .001) and more than a high school education (*P* < .001). Finally, diabetes prevalence increased significantly with higher BMI status (*P* < .001). Overweight adults were almost twice as likely to have diabetes than healthy-weight adults, and obese adults were nearly four times as likely than healthy-weight adults.

For the individuals in the study who had diabetes, self-reported age of diabetes diagnosis was assessed (data not shown). Among men who had diabetes, the average age of diagnosis for the three race/ethnicity categories was similar: 46.4 years (95% CI, 43.3–49.4) for Non-Hispanic whites, 45.1 years (95% CI, 41.4–48.8) for non-Hispanic blacks, and 45.0 years (95% CI, 42.1–47.9) for Mexican Americans. Overall, men with diabetes had a mean age of diabetes diagnosis of 45.7 years (95% CI, 43.2–48.3). In contrast, women who had diabetes showed more striking differences in age of diagnosis among race groups. The mean age of diagnosis was 48.8 years (95% CI, 44.6–53.0) for non-Hispanic white women, 43.6 years (95% CI, 41.6–45.6) for non-Hispanic black women, and 40.4 years (95% CI, 37.5–43.3) for Mexican American women. Overall, women who had diabetes had an average age at diagnosis of 46.4 years (95% CI, 43.9–49.0). In addition, individuals who had diabetes and were obese had a younger mean age of diabetes diagnosis at 43.7 years (95% CI, 40.9–46.6) than overweight (48.6 years; 95% CI, 45.8–51.4) and healthy-weight (47.3 years; 95% CI, 44.1–50.4) individuals with diabetes.

### Family history

Table 2 displays the frequencies and percentages of individuals who had diabetes in the study according to family history status: 3172 adult respondents reported having a family history of diabetes in a first-degree relative (parents and siblings) within the study population of 10,283. The diabetes prevalence for individuals with a family history was more than four times higher than the prevalence for individuals without a family history (*P* < .001). Among adults with a family history, diabetes prevalence increased significantly with a corresponding increase in number of family members with diabetes (*P* < .001). The diabetes prevalence for individuals with three or more first-degree relatives with diabetes (44.4%) was higher than the prevalence associated with any other demographic or risk factor measured.

Diabetes prevalence associated with parental history significantly increased with the number of affected parents (*P* < .001). The diabetes prevalence for individuals with a diabetic mother (16.5%) was higher than for individuals with a diabetic father (12.4%). In addition, having a sibling with diabetes conferred a diabetes prevalence approximately 4.5 times higher than the prevalence for individuals without a diabetic sibling (*P* < .001).

Further assessment of age of diagnosis (data not shown) showed that among individuals with diabetes who had a first-degree relative with diabetes, the mean age of diagnosis was 44.5 years (95% CI, 42.4–46.6) compared with 48.5 years (95% CI, 45.4–51.6) for individuals with diabetes who had no family history of diabetes. Moreover, there was more than an eight-year difference in mean age of diagnosis of individuals with diabetes whose parents had diabetes compared with individuals with diabetes whose parents did not have diabetes: 39.9 years (95% CI, 34.9–45.0) for individuals with two diabetic parents, 44.3 years (95% CI, 42.1–46.6) for individuals with one diabetic parent, and 48.3 years (95% CI, 45.7–51.0) for individuals with neither parent diabetic.

**Figure 1:**
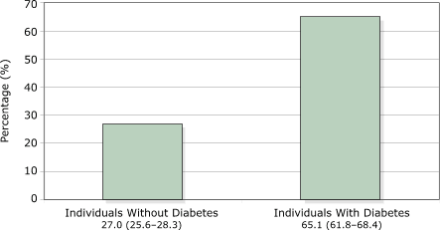
Percentages (95% confidence interval) of U.S. adults aged 20 years and older reporting a family history of diabetes, by self-reported diabetes status, NHANES 1999–2002.

**Figure 2:**
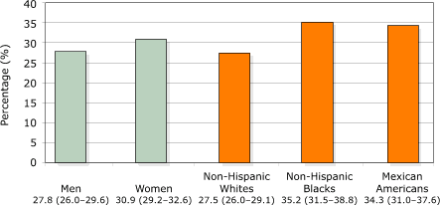
Percentages (95% confidence interval) of U.S. adults aged 20 years and older reporting a family history of diabetes, by gender and race/ethnicity, NHANES 1999–2002.

**Figure 3:**
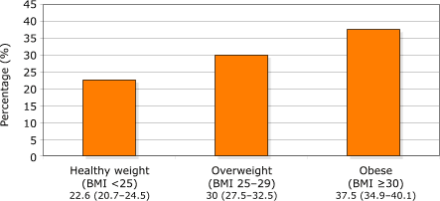
Percentages (95% Confidence IntervaI) of U.S. adults aged 20 years and older reporting a family history of diabetes, by body mass index (BMI), NHANES 1999–2002.

The presence of family history among adults differed by several factors and is depicted in Figures 1–3. A significantly larger proportion of individuals with diabetes reported having a family history of diabetes than individuals without diabetes (*P* < .001). More women reported a family history than men (*P* = .006). Compared with non-Hispanic whites, a higher percentage of non-Hispanic blacks (*P* = .001) and Mexican Americans (*P* < .001) reported a family history of diabetes. And obese and overweight adults were more likely to have a family history of diabetes than healthy-weight adults (*P* < .001 for both).

**Table:** 

**Table:** 

**Table:** 

### Multivariate analyses

The association of family history and diabetes was evaluated with four regression models shown in Table 3. Each model used a different variable for family history and analyzed these variables first independently (crude ORs), then with the addition of other demographic and risk factors in the model (adjusted ORs). The family history variable was statistically significant in crude analyses of each model. Adults with a family history of diabetes had five times the odds of having diabetes compared with individuals who did not have a family history of diabetes.

The adjusted models used the categorical factors of gender, age group, race/ethnicity, PIR, and BMI. Since PIR and education level were highly related, education level was not included in the models. Regression analyses were also performed using age, PIR, and BMI as continuous variables; however, this did not appreciably change the parameter estimates corresponding to family history. In each of the four models, all additional variables were statistically significant, with the exception of BMI 25–29, for which the β estimate had a *P* value of .051 (Model 1) and .052 (Model 2).

After adjusting for the other variables, family history remained significantly associated with diabetes status, though the adjusted ORs were slightly lower than the crude ORs. Adults with a family history of diabetes had four times the odds of having diabetes themselves compared with individuals without a family history (*P* < .001). The odds of having diabetes were almost 15 times higher for those with three or more diabetic relatives compared with adults with no family history (*P* < .001). Parental and sibling diabetes history were also significantly associated with increased risk of diabetes (*P* < .001 for both).

## Discussion

Our diabetes prevalence estimates for the gender–race groups were similar to a previous review of data from NHANES III (1988–1994), which showed that for both men and women, non-Hispanic blacks had a higher diabetes prevalence than non-Hispanic whites and Mexican Americans (37). However, we did not find any studies using NHANES data that examined family history of diabetes in relation to diabetes prevalence.

We found that family history of diabetes was a significant predictor of self-reported diabetes among U.S. adults. We estimated that adults with a family history of diabetes in a parent or sibling had four times the odds of having diabetes than adults without a family history of the disease, after adjusting for gender, age, race, PIR, and BMI. These findings are consistent with a recent summary review of 10 studies performed in various countries, which reported that individuals with a positive family history of diabetes had two to six times the risk of type 2 diabetes, compared with individuals without a family history of the disease (19).

Moreover, our study demonstrated that adults with two diabetic parents had more than twice the risk of diabetes than adults with only one diabetic parent. This additive risk association has been described previously in a white U.S. population (22). Through further examination of family history, an elevated diabetes risk was found to be associated with an increased number of first-degree family members affected with diabetes. Among all demographic and risk factors, the presence of three or more diabetic first-degree relatives corresponded to the highest diabetes prevalence and OR for diabetes. With the exception of a few studies, a relatively small amount of literature quantified family history of diabetes in terms of the number of affected relatives.

Because family history was one of the strongest risks for diabetes in our study, individuals with family members who have diabetes should be a screening priority for diabetes. As stated previously, undiagnosed diabetes constitutes approximately 29.3% of total diabetes prevalence (5). A current study demonstrated that the prevalence of diagnosed diabetes has increased, and the prevalence of undiagnosed diabetes has decreased for severely obese individuals (BMI ⩾35), possibly because of a better awareness of BMI as a risk factor among health care providers and improved screening among these individuals (5). Similarly, the use of a family history screening tool could capture many more of these undiagnosed individuals who would benefit from early intervention.

Individuals who have close relatives with diabetes may be more motivated to seek early health screening and thus more likely to be diagnosed than individuals without a family history. Because of earlier screening, individuals with a family history would likely be younger at age of diagnosis than individuals without a family history. This likelihood is supported by both our study (44.5 years at diagnosis for individuals with a family history vs 48.5 years at diagnosis for individuals without a family history) and an Australian study, which found a trend of younger age of diabetes diagnoses with increasing number of family members affected (24). Furthermore, research has shown that individuals with type 2 diabetes are more likely to collect health information from family members (38). However, our study indicated that a higher proportion of adults who had diabetes did not know their family history of diabetes (2.7%) when compared with adults who did not have diabetes (2.0%), although this difference was not statistically significant.

In addition, proportionately more women reported a father, mother, brother, or sister with diabetes than men, and there were more reports of female relatives with diabetes than male relatives with diabetes. A recent study found that women were slightly more likely than men to regard family history as very important to their own health and were more likely to collect family medical information (38). Among men in our study, 2.2% did not know their family history of diabetes, compared with 1.8% of women.

### Limitations

Limitations of our study include the inability to discriminate between cases of type 1 and type 2 diabetes. Had stratification been possible, we may have found different relationships among diabetes, family history, and other factors. Subjects in our study were not excluded based on age of diabetes diagnosis; such exclusion could have eliminated many type 1 diabetes cases. It is estimated that approximately one third of children with diabetes aged 12 to 19 years have type 1 diabetes. The prevalence of type 1 diabetes among all ages in the United States is approximately 0.12% (39). Therefore, the exclusion of individuals with type 1 diabetes from our study population would probably not have affected our results appreciably.

Because diabetes diagnoses of participants and family members were self-reported and not verified, the true diabetes prevalence may be misrepresented. Moreover, diabetes is underdiagnosed in the United States, suggesting that the true prevalence is higher than reported prevalence. Subjects also self-reported age of diabetes diagnosis, creating a potential for recall bias. As previously mentioned, survey participants were not asked about family history of diabetes in children, which limited our definition of first-degree relatives to parents and siblings only. Also, NHANES excludes institutionalized persons, including individuals residing in nursing homes, who are likely to be older adults.

### Implications

Our findings create several implications for public health. First, diabetes has paralleled the obesity epidemic. Similar to a previous NHANES study (40), we found that non-Hispanic black women had the highest prevalence of obesity (48.7%) compared with non-Hispanic white women (31.1%), Mexican American women (36.8%), non-Hispanic black men (26.8%), non-Hispanic white men (27.9%), and Mexican American men (25.8%). The prevalence of family history was also highest in women and non-Hispanic blacks among genders and races. Both obesity and diabetes have strong environmental components, such as diet and physical activity. Thus, the presence of family history often reflects the shared environment and health-related behaviors among family members in addition to hereditary factors. The recognition of this high correlation among obesity, diabetes, and family history can help guide population-appropriate health promotion activities.

Second, with the current striving for genetic awareness and competency in public health, this study represents a feasible and inexpensive method of extracting genomic information from existing population-based data sources. NHANES, a validated and well-recognized survey, provides a substantial amount of health information on a national level. Other population-based surveys also offer informative data that may pertain to genomics. There are several steps public health practitioners can take now to access and use genomics and incorporate genomics into programs. Because family history encompasses both genetic and environmental factors, it can be applied to other chronic diseases involving multiple complex etiologies, such as cardiovascular disease. Therefore, knowledge gained from family history and diabetes can be translated into other public health program areas.

Finally, at the primary care and public health level, this study supports the promotion of a family history tool for diabetes prevention and early detection strategies as a valuable measure of diabetes risk. Family history is easily available and inexpensive to obtain yet may be underused in health care practice (31). The following three criteria are suggested for incorporating a family history tool into public health screening: 1) the disease represents a significant public health burden, 2) family history is an established risk factor, and 3) there are effective interventions for prevention (31). Type 2 diabetes meets these criteria. It is evident that neither diabetes nor obesity prevalence is decreasing; therefore, it is critical that we use all available resources to quantify individual disease risk as accurately and completely as possible.

## Figures and Tables

**Table 1:** Frequencies and Percentages of Self-Reported Individuals With Diabetes by Demographic and Risk Factors, Adults Aged 20 Years and Older in the United States, 1999–2002

	**Total (n)**	**Diabetic (n)**	**Weighted^a^ % (95% CI)**
Total	10,283	991	6.5 (5.9-7.1)

**Men**			

All races	4802	481	6.7 (5.9-7.5)
Non-Hispanic white	2396	196	6.2 (5.1-7.3)
Non-Hispanic black	887	108	8.2 (6.5-9.9)
Mexican American	1129	130	5.3 (3.9-6.7)

**Women**			

All races	5481	510	6.3 (5.5-7.2)
Non-Hispanic white	2674	170	5.1 (4.4-5.8)
Non-Hispanic black	1034	140	11.4 (9.2-13.6)
Mexican American	1266	148	7.7 (6.2-9.3)

**Age, years**			

20-39	3618	61	1.7 (1.1-2.2)
40-59	2964	256	6.6 (5.6-7.5)
⩾60	3701	674	15.1 (13.9-16.4)

**Poverty income ratio (PIR)**			

PIR <1.00<(poverty)	1743	208	7.9 (6.0-9.8)
PIR 1.00-1.85	2138	278	8.7 (7.3-10.1)
PIR ⩾1.86	5222	378	5.3 (4.7-6.0)

**Education level**			

Less than high school	3559	514	10.9 (9.5-12.3)
High school/GED	2361	200	6.5 (5.4-7.5)
More than high school	4321	273	4.7 (3.8-5.5)

**Body mass index (BMI)^b^**			

BMI <25	2752	143	3.1 (2.3-3.9)
BMI 25-29	3087	298	5.9 (4.8-7.0)
BMI ⩾30	2662	386	11.2 (10.1-12.4)

a For extrapolation of diabetes prevalence to the adult, noninstitutionalized, civilian U.S. population, weighted percentages incorporate NHANES sampling weights to account for unequal selection probabilities and nonrandom sampling design.

b Excludes pregnant women.

**Table 2:** Frequencies and Percentages of Self-Reported Individuals With Diabetes by Family History of Diabetes, Adults Aged 20 Years and Older in the United States, 1999–2002

	**Total (n)^a^**	**Diabetic (n)**	**Weighted^b^ % (95% CI)**

**Family history of diabetes (parents and/or siblings only)**			

No	6895	344	3.2 (2.8-3.6)
Yes	3172	618	14.3 (12.8-15.9)

**Number of relatives with diabetes (parents and/or siblings only)**			

One relative	2343	354	11.0 (9.5-12.5)
Two relatives	606	148	19.3 (15.4-23.2)
Three or more relatives	223	116	44.4 (37.7-51.0)

**Parental history of diabetes**			

Neither parent has diabetes	7640	512	4.2 (3.7-4.7)
One parent has diabetes	2181	368	12.3 (10.7-13.9)
Both parents have diabetes	246	82	25.4 (18.8-31.9)
Father has diabetes	1046	177	12.4 (10.0-14.7)
Mother has diabetes	1627	355	16.5 (14.6-18.5)

**Sibling history of diabetes**			

No sibling has diabetes	8749	596	4.7 (4.2-5.2)
At least one sibling has diabetes	1318	366	21.7 (19.2-24.3)
Brother(s) has/have diabetes	756	228	23.3 (19.7-26.9)
Sister(s) has/have diabetes	828	257	25.6 (22.2-28.9)

a Family history status was not ascertained for 216 of the 10,283 participants in the National Health and Nutrition Examination Survey 1999–2002.

b For extrapolation of diabetes prevalence to the adult, non-institutionalized, civilian U.S. population, weighted percentages incorporate NHANES sampling weights to account for unequal selection probabilities and nonrandom sampling design.

**Table 3:** Odds Ratios and 95% Confidence Intervals for Diabetes by Family History, Adults Aged 20 Years and Older in the United States, 1999–2002^a^

**Model**	**Crude**		**Adjusted^b^**	

	**OR**	**95% CI**	**OR**	**95% CI**

**Model 1:Family history of diabetes (parents and/or siblings only)^c^**				

No	1.00 (ref)	NA	1.00 (ref)	NA
Yes	5.06	4.37-5.85	3.95	3.25-4.79

**Model 2:Number of relatives with diabetes (parents and/or siblings only)^c^**				

None	1.00 (ref)	NA	1.00 (ref)	NA
One relative	3.74	3.15-4.43	3.05	2.44-3.82
Two relatives	7.25	5.63-9.34	5.14	3.81-6.91
Three or more relatives	24.12	18.24-31.89	14.83	10.95-20.08

**Model 3:Parental history of diabetes^c^**				

No	1.00 (ref)	NA	1.00 (ref)	NA
One parent has diabetes	3.17	2.65-3.79	3.04	2.34-3.94
Both parents have diabetes	7.68	5.63-10.48	6.95	4.69-10.29

**Model 4: Sibling history of diabetes^c^**				

No	1.00 (ref)	NA	1.00 (ref)	NA
At least one sibling has diabetes	5.59	4.80-6.51	3.52	2.94-4.21

a OR indicates odds ratio; CI indicates confidence interval; ref indicates referent group; NA indicates not applicable.

b Full regression models are adjusted for gender (males, females), age group (20–39, 40–59, ⩾60 years), race/ethnicity (non-Hispanic white, non-Hispanic black, Mexican American, other), poverty income ratio (PIR) (PIR <1.00, PIR 1.00–1.85, PIR ⩾1.86), and body mass index (BMI <25, BMI 25–29, BMI ⩾30). Females, aged 20–39 years, non-Hispanic white, PIR ⩾1.86, and BMI <25 were used as referent groups

c Overall model significance: P < .001.
